# Telemedicine and Patient Experience Ratings at an Academic Integrative Medicine Practice: Retrospective Examination

**DOI:** 10.2196/56312

**Published:** 2024-07-22

**Authors:** Ellen Meltzer, Laurie Wilshusen, Isra Abdulwadood, Claire Yee, Amy Sherman, Kelli Strader, Barbara Thomley, Denise Millstine, Jon Tilburt, Heather Fields, Larry Bergstrom, David Patchett, John Camoriano, Brent Bauer

**Affiliations:** 1 Division of General Internal Medicine Department of Medicine Mayo Clinic Arizona Scottsdale, AZ United States; 2 Office of Experience Mayo Clinic Arizona Scottsdale, AZ United States; 3 Section of Integrative Medicine Mayo Clinic Arizona Scottsdale, AZ United States; 4 Mayo Clinic Quality Management Services Mayo Clinic Scottsdale, AZ United States; 5 Mayo Clinic Alix School of Medicine Scottsdale, AZ United States; 6 Quantitative Health Sciences Mayo Clinic Arizona Scottsdale, AZ United States; 7 Division of General Internal Medicine Section of Integrative Medicine Mayo Clinic Arizona Scottsdale, AZ United States; 8 Mayo Clinic Women’s Health Center Section of Integrative Medicine Mayo Clinic Arizona Scottsdale, AZ United States; 9 Mayo Clinic Community Internal Medicine Section of Integrative Medicine Mayo Clinic Arizona Scottsdale, AZ United States; 10 Mayo Clinic Family Medicine Section of Integrative Medicine Mayo Clinic Arizona Scottsdale, AZ United States; 11 Hematology/Oncology Section of Integrative Medicine Mayo Clinic Arizona Phoenix, AZ United States; 12 Division of General Internal Medicine Section of Integrative Medicine Mayo Clinic Rochester, MN United States

**Keywords:** telemedicine, TELE, patient experience, communication skills, integrative medicine, face-to-face, F2F, encounters

## Abstract

**Background:**

The use of telemedicine (TELE) increased exponentially during the COVID-19 pandemic. While patient experience with TELE has been studied in other medical disciplines, its impact and applicability to integrative medicine practices remain unknown.

**Objective:**

The aim of this study is to assess the impact of visit modality, TELE versus face-to-face (F2F) encounters, on patient experience at an integrative medicine practice at a single academic medical center. Given the significant role of the patient-physician relationship, therapeutic presence, and touch in integrative medicine, we hypothesized that TELE would result in reduced patient experience compared to traditional F2F encounters.

**Methods:**

A retrospective examination of Press Ganey surveys at an academic, consultative, and integrative medicine practice was conducted. Anonymous surveys completed by patients, older than 18 years of age, who had TELE or F2F appointments from April 1, 2020, to March 31, 2023, were included. At our medical center, patients commonly travel in from out of state for complex care. We examined percentage “top box” scores (ie, the percentage of respondents who selected the most positive response category on the survey, “very good”), across a variety of experience metrics. ANOVA and chi-square analyses were completed, with a significance threshold of *P*<.05.

**Results:**

Over the 36 months, a total of 1066 surveys were completed and returned (TELE: n=333; F2F: n=733). Overall, 73% (n=778) of respondents were female with an average age of 57.6 (SD 13.84) years. Most patients were English-speaking (n=728, 99.3%), White (n=1059, 92.7%), and not Hispanic or Latino (n=985, 92.4%). There was significantly higher satisfaction with access to care for TELE visits compared to F2F visits. There were no differences in satisfaction with the care provider or in overall experience. When examining the specific aspects of using technology during TELE visits, there were no differences in audio quality, visual quality, or ease of talking to the care provider based on sex. There was, however, a difference in video quality based on age, where those 80 years and older rated significantly lower video quality compared to all other age groups.

**Conclusions:**

Top-level patient experience can be attained with TELE integrative medicine visits. Additional studies, particularly those correlating positive experience findings with specific behaviors used during TELE visits, would further our understanding of the integrative medicine patient experience. In the meantime, efforts should be made to ensure a policy that promotes the ongoing provision of TELE in integrative medicine.

## Introduction

The use of telemedicine (TELE), the provision of health care through 2-way telecommunications, increased exponentially during the COVID-19 pandemic [1]. Many expect that these changes will persist beyond the COVID-19 pandemic [2]. Prior studies of TELE visits in various medical specialties have demonstrated relatively favorable patient satisfaction with this type of encounter [3-6]. The impact of TELE on patient experience in integrative medicine is less examined.

To address these concerns, this study assessed the impact of visit modality, TELE video visits versus face-to-face (F2F) encounters, on patient experience at an integrative medicine practice at a single academic medical center. Given the vital role of the patient-clinician relationship in integrative medicine, it was hypothesized that TELE might result in reduced patient experience compared to the traditional, F2F encounters.

Patient experience measures, as defined by the Agency for Healthcare Research and Quality, “encompasses the range of interactions that patients have with the health care system...” and, when positive, is associated with better clinical outcomes [5]. It is important to note that patient experience, as measured through publicly available surveys such as Press Ganey surveys, can be significantly impacted by TELE [7]. In particular, the relational aspects of care including verbal and nonverbal communication can be affected [8]. This is relevant for integrative medicine, which emphasizes whole-person care and views the therapeutic patient-clinician relationship, including touch and presence as central to the healing process [9]. Appropriately, there is concern that TELE can adversely affect the integrative medicine patient experience [8,9].

## Methods

### Study Design and Respondents

The study team conducted a retrospective examination of anonymous, validated outpatient, patient experience surveys at a consultative, academic, and integrative medicine practice. Respondents were patients older than 18 years who had TELE or F2F appointments from April 1, 2020, to March 31, 2023, in integrative medicine. At our medical center, patients commonly travel in from out of state for complex care. Patients seen in integrative medicine generally have 1-2 visits with the integrative medicine clinician who, in partnership with the patient, develops an integrative care plan that the patient may follow with the support of their local, primary care provider. Both TELE and F2F appointments are offered. The practice has a total of 7 physicians (3 male and 4 female, with an average of 8.5 years practicing integrative medicine, who also practice internal medicine, family medicine, hematology or oncology, and women’s health) and uses 1 shared, digital video platform [10]. The study was conducted in accordance with SQUIRE 2.0 guideline (Standards for Quality Improvement Reporting Excellence).

### Ethical Considerations

The Mayo Clinic institutional review board deemed the study exempt from further review.

### Patient Experience

Patient experience was assessed using 3 scales from The Press Ganey Medical Practice Survey. The survey is one of the most commonly used surveys for measuring patient experience with outpatient health care and is sent to patients after an outpatient visit [10]. The access subscale consisted of 2 items (eg, ease of getting through to the clinic on the phone) measuring accessibility to care. The care provider subscale consisted of 10 items (eg, concern the care provider showed for your questions or worries) assessing satisfaction with the care provider specifically. Finally, the overall assessment subscale included 2 items measuring overall patient experience (eg, the likelihood of your recommending our practice to others). All items were answered on a scale of 1 (very poor) to 5 (very good). Each subscale was calculated as a mean from all items in the subscale. All 3 subscales showed high internal reliability (Cronbach α=0.78-0.97).

### Technology Experience

A total of 3 general items measured the quality of the TELE visits (eg, how well the audio connection worked during your visit). The items were assessed on a 1 (very poor) to 5 (very good) scales.

### Statistical Analysis Plan

Descriptive statistics were calculated using mean and SD for continuous variables, and frequencies and percentages for categorical variables. Independent samples *t* tests examined differences in continuous variables between TELE and F2F visits, while chi-square tests were used to assess for potential differences in categorical variables. Independent samples *t* tests and 1-way ANOVAs evaluated differences in demographic factors and technology experience. All analyses used a significance threshold of *P*<.05. Analyses were conducted in SAS (version 9.04; SAS Institute).

## Results

Over the 36 months, a total of 1066 surveys were completed and returned (TELE: n=333; F2F: n=733). Overall, 73% (n=778) of respondents were female with an average age of 57.6 (SD 13.84) years. Most patients were English-speaking (n=1059, 99.3%), White (n=988, 92.7%), and not Hispanic or Latino (n=985, 92.4%; Table 1). Differences were observed in race and ethnicity (Table 1). For race, these differences appeared to be driven primarily by other and unknown categories. There was slightly more variation in proportions for the TELE visits compared to the F2F visits, which were primarily not Hispanic or Latino. TELE visits also had a higher proportion of commercial insurance payers. No other differences existed between the 2 visit types in participant demographics. There was significantly higher satisfaction with access to care for TELE visits compared to F2F visits. There were no differences in satisfaction with the care provider or overall experience (Table 2).

When examining the specific aspects of using technology during TELE visits, there were no differences in audio quality, visual quality, or ease of talking to the care provider based on sex. There was, however, a difference in video quality based on age, where those 80 years and older, rated significantly lower video quality compared to all other age groups (Table 3).

**Table 1:** Demographics of patients.

Variable			Visit type					Total (N=1066)			*P* value
			F2F^a^ (n=733)		TELE^b^ (n=333)						
Age (years), mean (SD)			57.7 (14.3)		57.5 (12.7)		57.6 (13.8)			.90^c^	
**Sex, n (%)**											.99^d^
	Female	535 (73.0)		243 (73.0)		778 (73.0)					
	Male	198 (27.0)		90 (27.0)		288 (27.0)					
**Race, n (%)**											.003^d^
	American Indian or Alaska Native	1 (0.1)		7 (2.1)		8 (0.8)					
	Asian Indian	5 (0.7)		2 (0.6)		7 (0.7)					
	Black or African American	14 (1.9)		6 (1.8)		20 (1.9)					
	Chinese	2 (0.3)		0 (0.0)		2 (0.2)					
	Korean	2 (0.3)		0 (0.0)		2 (0.2)					
	Native Hawaiian	0 (0.0)		2 (0.6)		2 (0.2)					
	White	689 (94.0)		299 (89.8)		988 (92.7)					
	Other Asian	5 (0.7)		1 (0.3)		6 (0.6)					
	Unknown	6 (0.8)		7 (2.1)		13 (1.2)					
	Other	9 (1.2)		9 (2.7)		18 (1.7)					
**Ethnicity, n (%)**											.002^d^
	Central American	3 (0.4)		3 (0.9)		6 (0.6)					
	Hispanic or Latino	16 (2.2)		15 (4.5)		31 (2.9)					
	Mexican	2 (0.3)		0 (0.0)		2 (0.2)					
	Not Hispanic or Latino	692 (94.4)		293 (88.0)		985 (92.4)					
	Other Spanish culture	0 (0.0)		3 (0.9)		3 (0.3)					
	Puerto Rican	2 (0.3)		7 (2.1)		9 (0.8)					
	South American	2 (0.3)		0 (0.0)		2 (0.2)					
	Unable to provide	1 (0.1)		1 (0.3)		2 (0.2)					
	Unknown	1 (0.1)		0 (0.0)		1 (0.1)					
	Choose not to disclose	14 (1.9)		11 (3.3)		25 (2.3)					
**Language, n (%)**											.47^d^
	Arabic	1 (0.1)		0 (0.0)		1 (0.1)					
	English	728 (99.3)		331 (99.4)		1059 (99.3)					
	Korean	2 (0.3)		0 (0.0)		2 (0.2)					
	Norwegian	0 (0.0)		1 (0.3)		1 (0.1)					
	Spanish	2 (0.3)		1 (0.3)		3 (0.3)					
**Insurance, n (%)**											.001^d^
	Commercial	381 (54.4)		201 (60.5)		582 (56.4)					
	Medicare or Medicaid	252 (36)		104 (31.3)		356 (34.5)					
	Other	67 (9.6)		27 (8.1)		94 (9.1)					
	Missing	33 (0.05)		1 (0.01)		34 (0.03)					

^a^F2F: face-to-face.

^b^TELE: telemedicine.

^c^*P* value obtained from independent samples *t* test.

^d^*P* value obtained from chi-square test.

**Table 2:** Patient experience by visit type: F2F^a^ versus TELE^b^.

Measure	Visit type, mean (SD)		Total (N=1066), mean (SD)		*P* value
	F2F (n=733)	TELE (n=333)			
Access	4.5 (0.73)	4.7 (0.5)	4.6 (0.68)	<.001	
Care provider	4.7 (0.65)	4.8 (0.56)	4.8 (0.62)	.80	
Overall	4.8 (0.67)	4.7 (0.64)	4.8 (0.66)	.60	

^a^F2F: face-to-face.

^b^TELE: telemedicine.

**Table 3:** Patient experience of technology experience measures for TELE^a^ visits.

		How well the audio connection worked during your visit	Ease of talking with the care provider	How well the video connection worked
Total (N=333), mean (SD)		4.7 (0.56)	4.7 (0.61)	4.8 (0.50)
**Sex, mean (SD)**				
	Female (n=243)	4.7 (0.56)	4.8 (0.61)	0.8 (0.50)
	Male (n=90)	4.7 (0.58)	4.7 (0.71)	4.8 (0.59)
	*P* value	.13^b^	.26^b^	.58^b^
**Age (years)** **,** **mean (SD)**				
	<35 (n=16)	4.8 (0.45)	4.8 (0.40)	4.7 (0.48)
	35-49 (n=70)	4.7 (0.51)	4.7 (0.64)	4.7 (0.69)
	50-64 (n=132)	4.7 (0.66)	4.7 (0.64)	4.8 (0.47)
	65-79 (n=106)	4.8 (0.44)	4.7 (0.67)	4.9 (0.39)
	≥80 (n=9)	4.2 (0.83)	4.7 (0.50)	4.3 (0.87)
	*P* value	.07^b^	.90^b^	.02^b^

^a^TELE: telemedicine.

^b^*P* value obtained from independent samples *t* test.

## Discussion

To better understand and improve the experiences of integrative medicine patients, this study assessed associations between visit modality (TELE vs F2F encounters) and patient experience measures. Despite the significant role of the patient-clinician relationship in integrative medicine and the role of touch and presence, the hypothesis that TELE would be associated with lower patient experience compared to traditional, F2F encounters did not bear out. On the contrary, these exploratory data suggest high-level patient experience scores can be achieved at a level comparable to F2F encounters, for consultative integrative TELE.

Integrative medicine emphasizes the relational aspects of care, and prior studies of TELE in other patient populations have highlighted concerns that patient experience and, specifically, the patient-clinician relationship can be negatively impacted by TELE. TELE has been previously described as “impersonal” [8]. Researchers have noted a decrease in verbal expressions of empathy during TELE encounters [11,12], and understandably, TELE limits clinician opportunities to express empathy through nonverbal communication skills, such as touch [8,13-16]. Interestingly, despite these potential limitations, physicians in this study achieved top-level TELE patient experience scores across all care provider metrics, including “Concern the care provider showed for your questions or worries.” While this study did not specifically evaluate physician communication skills, prior experience research completed by members of this study team demonstrates that implementing empathic, patient-centered communication improves patient experience [17], and it is well known that empathic communication skills can be taught [18]. All physicians who provided clinical care to patients in this study completed an in-person, 8-hour course in empathic F2F communication as a part of their orientation during onboarding. While this training was prior to the COVID-19 pandemic and did not include instruction in empathic communication during TELE visits, it still may have positively influenced the TELE patient experience as indicated by these data. An important question remains about which communication skills truly correlate with a better patient TELE experience. Additional studies, particularly ones in which top-performing TELE clinicians are observed, could serve to identify best communication practices for TELE visits.

With these favorable preliminary data, it is then important to ask, when is TELE particularly useful for integrative medicine? A recent systematic review of 44 TELE studies suggests that TELE can improve clinical outcomes, communication, and access to care [19]. It is known that lack of in-person access to integrative medicine can contribute to integrative health disparities [20] and with growing comfort and availability of integrative medicine TELE visits, there is a real opportunity to increase access to experts in integrative medicine; however, this is not without concerns. Some argue that digital resource limitations and lack of access to TELE can perpetuate disparities, rather than alleviate them [20-22]. Others suggest that TELE adoption may also be limited among some older adults [23]. This study did not examine whether any older patients might have, in advance of the appointment, changed from TELE to F2F based on personal preference. The older adults in this study, however, did report positive experiences similar to that of younger patients.

This exploratory study has limitations. Data were collected at a single academic medical center in Arizona with a largely White, college-educated patient population and, in this sample, a high race concordance between patient and physician. These results might not be readily generalizable to other patient populations. Replicating this study in other, more diverse integrative medicine clinical practices would significantly contribute to understanding patients’ experiences with, and barriers to, integrative TELE. In addition, our data did not include metrics on “wait time” for patients with TELE visits, only for those with F2F visits. For F2F visits, 87% (n=637) of patients selected the top box score for wait time. As prior research has suggested that TELE visits can serve to reduce wait time, thus improving the experience, future studies should include this measure [24]. Finally, the relatively small sample size biased results toward the null. As TELE integrative medicine grows, larger studies are needed. Finally, our study does not distinguish between “new” and “return” patient visits, which may serve to impact patient experience with TELE visits [25].

In conclusion, top-level patient experience can be attained with TELE integrative medicine visits, suggesting TELE can facilitate access to integrative medicine for those who live in areas with fewer integrative clinicians. In addition, for patients who prefer not to leave their homes for medical visits, TELE helps to facilitate compliance with ongoing medical care. Correlating positive experience findings with specific behaviors used during TELE visits would further our understanding of the integrative medicine patient experience. In the meantime, efforts should be made to ensure a policy that promotes the ongoing provision of TELE in integrative medicine.

## Abbreviations

F2F: face-to-face
SQUIRE: Standards for Quality Improvement Reporting Excellence
TELE: telemedicine
